# Detection of Pig Cells Harboring Porcine Endogenous Retroviruses in Non-Human Primate Bladder After Renal Xenotransplantation

**DOI:** 10.3390/v11090801

**Published:** 2019-08-29

**Authors:** Yoonki Heo, Yeondong Cho, Keon Bong Oh, Ki Hoon Park, Hansam Cho, Hanul Choi, Minjee Kim, Ik Jin Yun, Hee Jung Lee, Young Bong Kim

**Affiliations:** 1 Department of Biomedical Science and Engineering, Konkuk University, Seoul 05029, Korea; 2 Department of Bio-industrial technologies, Konkuk University, Seoul 05029, Korea; 3 Animal Biotechnology Division, National Institute of Animal Science, RDA, Jeonju, Jeollabuk-do 54875, Korea; 4 Department of Surgery, Konkuk University Medical Center, Konkuk University School of Medicine, Seoul 05029, Korea

**Keywords:** pig-to-NHP xenotransplantation, heart xenotransplantation, kidney xenotransplantation, porcine endogenous retrovirus (PERV), microchimerism

## Abstract

Pigs are used as potential donor animals for xenotransplantation. However, porcine endogenous retrovirus (PERV), shown to infect both human and non-human primate (NHP) cells in vitro, presents a risk of transmission to humans in xenotransplantation. In this study, we analyzed PERV transmission in various organs after pig-to-NHP xenotransplantation. We utilized pig-to-NHP xenotransplant tissue samples obtained using two types of transgenic pigs from the National Institute of Animal Science (NIAS, Republic of Korea), and examined them for the existence of PERV genes in different organs via PCR and RT-PCR with specific primers. To determine PERV insertion into chromosomes, inverse PCR using PERV long terminal repeat (LTR) region-specific primers was conducted. The PERV gene was not detected in NHP organs in cardiac xenotransplantation but detected in NHP bladders in renal xenotransplantation. The insertion experiment confirmed that PERVs originate from porcine donor cells rather than integrated provirus in the NHP chromosome. We also demonstrate the presence of pig cells in the NHP bladder after renal xenotransplantation using specific-porcine mitochondrial DNA gene PCR. The PERV sequence was detected in the bladder of NHPs after renal xenotransplantation by porcine cell-microchimerism but did not integrate into the NHP chromosome.

## 1. Introduction

Xenotransplantation is an excellent alternative tool for replacing organs and resolving the issue of organ shortage for transplantation in patients with terminal organ failure [1,2,3,4]. Genetically modified pigs provide an important organ source in the development of xenotransplantation for the treatment of diabetes mellitus (pancreatic islets), heart, and kidney disfunction [5,6]. Initial encouraging results using porcine islets were obtained in non-human primate (NHP) models in New Zealand. Several groups have reported pig islet transplantation in diabetic NHPs that successfully maintained normoglycemia for periods >1 year. Life-supporting (orthotopic) kidneys from pigs expressing a single human complement regulatory protein have been shown to function for up to 90 days, and the survival of a non-life-supporting heart in the abdomen (heterotopic) has been documented to be >2 years [5].

However, immunological barriers must be overcome for cross-species transplantation, such as transplant rejection and porcine viral transmission. Porcine endogenous retroviruses (PERV) are proviral elements that may replicate in human cells with the risk of transmission in the setting of xenotransplantation [7,8]. PERV-A, -B, and recombinant PERV-A/C have been shown to infect both human and pig cells in vitro while PERV-C infection is mainly restricted to pigs [9]. Therefore, PERV transmission to humans presents a potential threat in the field of xenotransplantation and raises major concerns. A recent study reported that PERV-A/C adapted to human cells can infect NHP cells due to mutations in long terminal repeats that play an important role in viral replication [10]. Moreover, cases of PERV infection and viral gene expression were reported following transplantation of pig islet cells into immunodeficient animals or non-obese diabetic/severe combined immunodeficiency (NOD/SCID) mouse models [11,12]. PERV DNA and RNA were additionally detected at multiple points in transgenic mice expressing human PERV-A receptor 2, indicating virus replication after xenotransplantation [13,14,15].

Xenografting of porcine organs is known to cause zoonotic infections but the frequency of viral transmission from xenograft sources in host animals has not been established. Furthermore, little is known about the potential risk of infection in host animals treated with transient immunosuppressive agents [16]. In order to prevent acute immune rejection, a large amount of immunosuppressive drug is prescribed and continuous administration is required. Although long-term survival has become possible with the prescription of immunosuppressive drugs after interspecies transplantation, it has the risk of chronic immunosuppression-related infection, xenograft rejection, and host adaptive virus production [17,18,19,20,21].

Transplantation of porcine organs into NHP recipients leads to the major problem of hyperacute and acute rejection. Pre-formed antibodies against the alpha 1,3-galactosyltransferase (GT) epitope activate the complement system, resulting in rapid destruction of the xenograft and consequent hyperacute rejection [22,23]. *GGTA1* gene knockout pig heart expressing GT transplanted into NHP underwent acute, but not hyperacute, rejection [24]. Complement regulators, such as membrane cofactor proteins (MCP or CD46), CD55, and CD39, play a critical role in inhibiting complement activation, and may thus be useful in preventing acute rejection. Nucleotides such as adenosine triphosphate (ATP) and adenosine diphosphate (ADP) present in the blood are sequentially degraded into adenosine monophosphate (AMP) and adenosine by CD39 on the surface of vascular endothelial. This adenosine is known to be an important signaling factor that regulates thrombus formation and inflammatory responses. Indeed, studies have been reported to inhibit rejection in xenotransplantation by human CD39 overexpression [25,26,27]. Xenotransplantation of organs using a transgenic pig model with a knockout of *GGTA1* and expressing a complement regulator has been shown to effectively suppress the immune rejection response [28,29,30].

In the current investigation, we examined tissue samples from NHPs transplanted with organs of GT knockout transgenic pigs expressing MCP or CD39 to determine whether PERV is transmitted to host tissues after xenotransplantation.

## 2. Materials and Methods 

### 2.1. Animal and Tissues 

Tissues of xenotransplanted NHP (*n* = 4) and donor pigs were provided by the National Institute of Animal Science (NIAS, Republic of Korea). Rhesus macaques (*Macaca mulatta*) were used as xenograft recipients (Table 1). Heart transplantation was performed using organs from transgenic pig models with GT inactivation and MCP expression [31,32] or GT knockout and human CD39 expression [33]. The recipient NHP23-16′s tissues were isolated at 60 days after transplantation and NHP20-01 tissues were isolated at 18 days. For kidney transplantation, transgenic pigs with GT knockout and human CD39 expression were used. Tissues were isolated from NHPs at 25 and 35 days after transplantation. All tissue samples were frozen at 80 ℃ until required. This experiment was approved by the Orient Genia Institutional Animal Care and Use Committee (IACUC No. ORIENT-IACUC-16141, permitted on July 11, 2016).

### 2.2. Xenotransplantation

For heart xenotransplantation, the donor pig’s ascending aorta is attached to the abdominal aorta of the NHP, and the donor pig’s pulmonary artery to the inferior vein of the NHP. The porcine coronary arteries are perfused in the abdominal aorta, and the coronary venous blood enters the right heart through the coronary arteries and is then ejected into the inferior cava via the pulmonary trunk [34]. For kidney xenotransplantation, the donor pig’s kidney containing arteries, veins, and ureter are removed near the bladder. After the right kidney of the NHP is removed, the NHP’s aorta and the donor’s renal artery, the inferior vena of NHP and the donor’s renal vein are connected. Next, the ureter of the transplanted kidney is connected to the NHP’s bladder. Finally, the state of NHP was observed, and transplanted organs were excised

### 2.3. PCR and RT-PCR Detection of PERV Genes 

Genomic DNA was isolated from different tissues using a DNA mini kit (Qiagen, Valencia, CA, USA) and 50 ng of gDNA was used for PCR. Total RNA was isolated using a RNeasy mini kit (Qiagen, Valencia, CA, USA), according to the manufacturer’s instructions. cDNA was synthesized from 1 µg of RNA using SuperScript II reverse transcriptase (Invitrogen, Carlsbad, CA, USA), and was RT-PCR performed using specific primers for conserved PERV *gag* and *pol* (Table 2) [35]. The cycling reaction was performed under the following conditions: denaturation at 94 ℃ for 5 min, 30 cycles at 94 ℃ for 20 sec, 55 ℃ for 20 sec, 72 ℃ for 20 sec, and extension at 72 ℃ for 10 min. The housekeeping gene 18s rRNA was used for normalization of PCR data. 

### 2.4. Quantitative Real-Time PCR Analysis of PERV Genes 

The plasmid clones of the PERV *pol* gene were generated to serve as a quantitative standard. The PERV *pol* gene was amplified by RT-PCR from PK-15 (porcine kidney-15) cells. The PCR products were cloned into a pGEM-T easy vector (Promega, Madison, WA, USA) and sequenced by sequencing analysis (Macrogen, Seoul, Republic of Korea). The, concentration of the plasmid was measured using a spectrophotometer (Epoch, Biotek, Winooski, VT, USA), and this value was used to determine the copy number. Quantitative real-time PCR was conducted based on specific primers for the PERV pol gene using SYBR green (Takara, Seoul, Korea). The gDNA and cDNA of NHP20-06 were used for qRT-PCR under the same condition as PCR and RT-PCR. The PCR reaction was performed in a StepOnePlus real-time PCR system from Applied Biosystems (Foster City, CA, USA) with the following thermal cycle conditions: 10 min of pre-incubation at 94 ℃ and 40 cycles in three steps each (94 ℃ for 20 sec, 55 ℃ for 20 sec, 72 ℃ for 20 sec). The results were analyzed with StepOneplus software (Applied Biosystems, Foster City, CA, USA).

### 2.5. Analysis of PERV Integration

We analyzed the PERV insertion site in NHP bladders via inverse PCR to determine whether the virus was transmitted to the organ. The gDNA (200ng) of NHP bladders and donor pig kidneys were digested using Sau3AI (New EnglandBiolabs, Ipswich, MA, USA) and self-ligated with T4 DNA ligase (Promega, Madison, WA, USA). Next, fragments containing the PERV-host DNA junctions were amplified via PCR using PERV LTR primer 1 (Table 2). PCR products were re-amplified using PERV LTR primer 2 (Table 2). All reactions were performed over 35 cycles under the following conditions: 94 ℃ for 5 min, 30 sec at 94 ℃, 30 sec at 55 ℃, 20 sec at 72 ℃, and 72 ℃ for 10 min. The amplified products were cloned into pGEM-T easy vector (Promega, Madison, WA, USA) and DNA sequencing was performed by Macrogen Inc. (Seoul, Korea). Sequences immediately adjacent to PERV LTRs were aligned using the NCBI BLASTn program (National Center for Biotechnology Information, Bethesda, MD, USA).

### 2.6. Analysis of Porcine Cell Microchimerism using PCR 

A previous study reported a species-specificity molecular approach based on the amplification of the mitochondrial *cytochrome B* gene [36]. To determine the porcine cell microchimerism, we detected pig *cytochrome B* mitochondrial gene from porcine cell using PCR. The PCR mixture contained each 2.5mM dNTPs, 10 pmol of primers (Table 2), rTaq DNA polymerase (Takara, Seoul, Republic of Korea), and 50 ng of gDNA in a total volume of 20 µL. The PCR was performed for 30 cycles under the following condition: 94 ℃ for 5 min, 15 sec at 94 ℃, 15 sec at 60 ℃, 15 sec at 72 ℃, and 72 ℃ for 10 min. We confirmed the detection of *cytochrome B* gene using electrophoresis.

## 3. Results

### 3.1. PERV Is not Detected in Recipient NHPs of Heart Xenotransplantation

**Figure 1:**
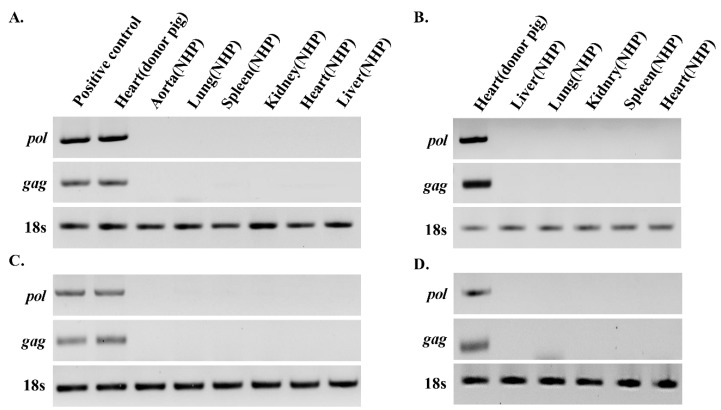
Analysis of PERV in different tissues of the heart transplanted recipients by PCR. The PCR and RT-PCR were performed to detect PERV *gag* and *pol* in different of heart transplanted recipients. The PERV level identified via electrophoresis and normalized to that of 18s rRNA. (**A**) gDNA and (**C**) cDNA of the heart transplanted recipients NHP 23-16. (**B**) gDNA and (**D**) cDNA of the heart transplanted recipients NHP 20-01.

Heart, aorta, lung, spleen, kidney, and liver tissue were sampled from the heart transplant recipient 23-16. Genomic DNA and RNA were isolated from these tissues and PCR was applied to detect PERV (Figure 1A,C). The *gag* and *pol* genes of PERV were detected in the heart of the GT-MCP/-MCP donor pig. Sampling was performed on the day of death after xenotransplantation. PERV *pol* and *gag* were not detectable in the heart, aorta, lung, spleen, kidney, and liver tissues of the host NHPs 23-16. Similarly, genomic DNA and RNA were isolated from heart, pulmonary, spleen, kidney, and liver tissues of the heart transplant recipient NHP 20-01 and PCR was employed to detect *pol* and *gag* genes of PERV (Figure 1B,D). Notably, PERV *pol* and *gag* were detected in porcine GT-CD39/-CD39 heart, but not recipient organs, clearly indicating that PERV from transplanted pig heart is not transmitted to recipient NHPs.

### 3.2. PERV Is Detected in Recipient NHPs of Kidney Xenotransplantation

**Figure 2:**
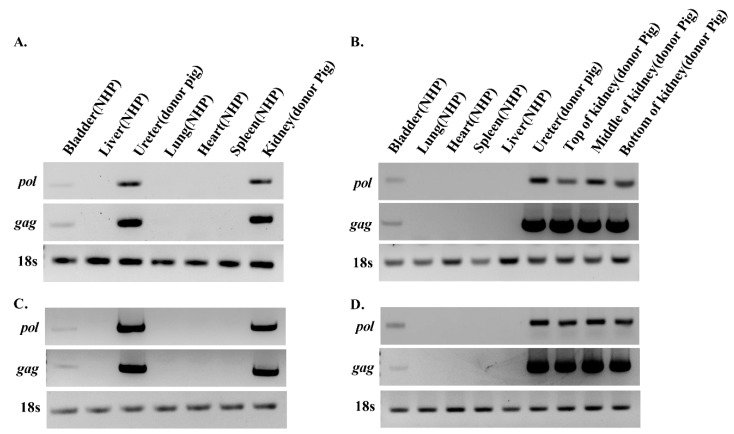
Analysis of PERV in different tissues of the kidney transplanted recipients by PCR. The PERV *gag* and *pol* were detected by PCR and RT-PCR from different tissues of the kidney transplanted recipients. (**A**) gDNA and (**C**) cDNA of the kidney transplanted recipients NHP 20-06. (**B**) gDNA and (**D**) cDNA of the kidney transplanted recipients NHP 23-30.

Heart, aorta, lung, spleen, kidney, and liver tissue were sampled from kidney transplant recipients NHP20-06 and NHP 23-30. Genomic DNA and RNA of the heart, bladder, liver, lung, ureter, spleen, and kidney were isolated from recipient NHPs as well as donor porcine GT-CD39/-CD39 kidney and ureter. PERV transmission was analyzed via PCR (Figure 2). In the case of NHP 20-06 (Figure 2A,C), *pol* and *gag* genes of PERV were not detected in the heart, lung, spleen, and liver tissue of the host animal. Interestingly, *pol* and *gag* were detected in renal and ureter tissue from NHPs, albeit at lower levels than donor tissues.

In another kidney transplantation case, NHP 23-30, genomic DNA and RNA were isolated from heart, bladder, liver, lung, ureter, spleen, and kidney, followed by PCR to detect PERV. Similar to NHP 20-06, *pol* and *gag* genes of PERV were not identified in the heart, lung, spleen, and liver, but detected in bladder tissue of the host animal.

Previously, we developed the classification methods for PERV types based on PCR using specific primers [35]. To identify the origin of PERVs, the *env* of PERV from both donors and recipients were detected by PCR using PERV types specific primers (Table S1). All three subtypes of PERV were confirmed in both gDNA of donor and recipients (Figure S1). Sequence analysis showed that the PERV sequences isolated from the recipient and donor were identical.

### 3.3. Quantitative Real-Time PCR Analysis of PERV

**Figure 3:**
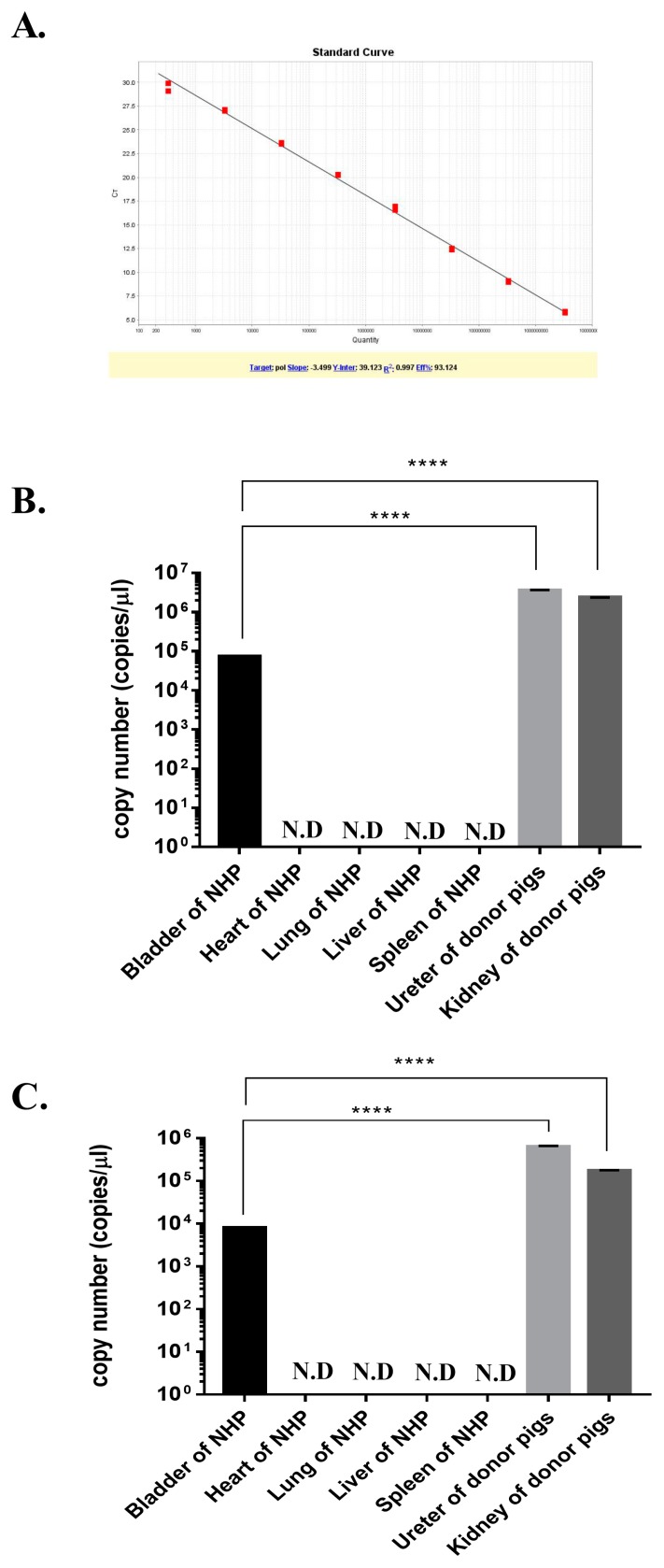
Quantitative analysis of PERV *pol* gene by real-time PCR. Quantitative real-time PCR was performed to detect the *pol* gene of PERV in tissues. (**A**) A typical standard curve derived from a serial dilution of standard plasmid DNA. (**B**) Copies/µL of PERV *pol* in gDNA, (**C**) Copies/µL of PERV *pol* in cDNA. Values are presented as means ± SD based on three tissues. (**** *p* < 0.0001 for comparison between the two groups).

PERV *pol* genes were detected in NHPs transplanted with porcine kidney. A quantitative real-time PCR was performed with 10-fold serial dilution of the standard plasmid DNA and the NHP tissues samples. The standard showed a strong linear relationship (*r*^2^ = 0.997), as shown in Figure 3A. The 33 copies/ µL were the detection limits of real-time PCR (more than 35 *C_T_* value or not detectable). Genomic DNA and cDNA of the bladder, heart, lung, liver, spleen, ureter, and kidney from recipient 20-06 were used. The number of PERV copies/ µL of NHP bladder gDNA and cDNA were 71,701 and 8056, respectively (Figure 3B,C). Compare to donor porcine organs (kidney and ureter), PERV DNA and RNA levels in the NHP bladder were 32.6–48.1 and 21.6–78.1 times lower, respectively.

### 3.4. Detection of PERV Is Not Due to Integration into the NHP Chromosome but to the Presence of Porcine Cells

**Figure 4:**
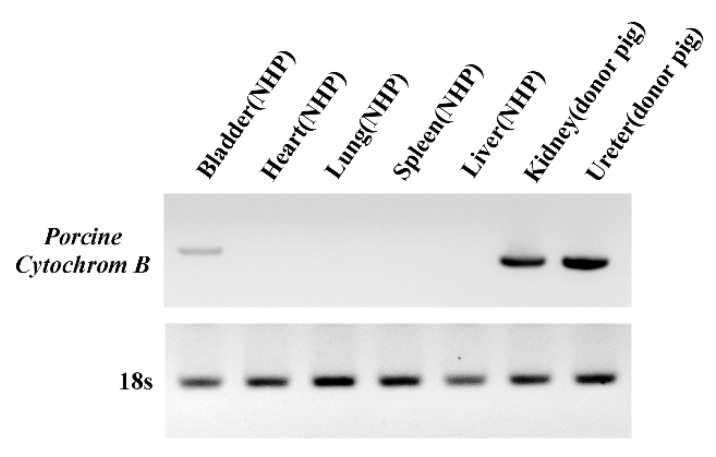
PCR analysis of porcine *cytochrome B* mitochondrial genes in different tissues of the kidney transplanted recipients for microchimerism determination. To determine the porcine cell microchimerism, we detected porcine *cytochrome B* mitochondrial genes in tissue using PCR. We confirmed the PCR product via electrophoresis.

Using the long-terminal repeat (LTR) region, integration of PERV into the host genome was analyzed via inverse PCR using specific primers. After shotgun cloning, inverse PCR-amplified genes were cloned and insert sequences analyzed. All cloned PERV LTR clones were derived from the donor porcine chromosome and no insertion into the NHP chromosome was detected (Table 3). Our data may indicate that PERV in the bladder represents circulating viruses or cells from the transplanted porcine kidney. To identify the microchimerism, we detected the porcine *cytochrome B* mitochondrial gene in gDNA. The porcine cytochrome B was not detected in heart, lung, spleen, and liver of NHP, but detected in NHP’s bladder, donor pig’s kidney, and donor pig’s ureter (Figure 4). The level in the NHP’s bladder was lower than that of donors and the porcine cells from transplanted porcine kidney were found to be present in the NHP’s bladder.

## 4. Discussion

Xenotransplantation using porcine organs is becoming a realistic strategy for the prevention and treatment of organ failure. Several clinical trials have been performed on islet transplantation for the treatment of diabetes and ex vivo perfusion using pig spleen or liver. To our knowledge, while PERV transmission has not been observed in preclinical and clinical xenotransplantation trials performed to date, the risk remains to be established [37].

To prevent immunological rejection, humanized pigs were developed and immunosuppressive drugs administered after transplantation [38]. The immunologically modified porcine organs are more likely to produce more infectious PERV in humans or NHPs. A previous report by our group showed PERV transmission from mice transplanted with mouse-adapted PERV-producing cells. In addition, the frequency of PERV transmission was increased in CsA-treated mice transplanted with PERV-producing murine cells, compared to PERV-producing porcine cells [21].

In the current study, transgenic porcine kidney and heart were transplanted into NHPs. Among the heart transplant tissues, PERV was detected in transplanted pig hearts, but not in other organs of NHP, indicating that PERV present in transplanted pig heart does not spread to other recipient tissues through the blood. The observed lack of PERV suggests lower risk of viral transmission in the case of heart xenografts. 

Following kidney transplantation, PERV was detected in the donor kidney and ureter as expected. However, PERV was identified in the bladder of NHPs. In addition, normalized to 18s rRNA, PERV levels of each tissue were compared. PERV levels in the bladder were lower than those in the donor kidney and ureter (Figure 2), and compared to the pig kidney and ureter, the bladder PERV copies/ µL of NHPs were lower than those of the donors (Figure 3). In view of these results, we suggest that lower levels of PERV gDNA and mRNA in the host bladder may be attributed to the transmission from donor kidney and ureter. To establish PERV transmission into host NHPs, the PERV integration test was conducted. We observed that the outer DNA sequence of PERV LTRs corresponded to the pig genome (Table 3). The porcine *cytochrome B* mitochondrial gene was detected in NHP’s bladder. These results suggest that porcine cells from donor pig tissue form microchimerism in the NHP bladder (Figure 4). Similar to our results, there are reports that PERVs were detected in peripheral blood mononuclear cell (PBMC) of NHPs after cardiac and renal xenotransplantation by cell microchimerism [39,40]. Our data show that despite the presence of PERV in the bladder, the virus was not integrated into NHP chromosomes, indicating that PERV particles and pig cells are non-infective circulating urinary vessels.

However, one major concern is that NHPs (baboons, rhesus monkeys, cynomolgus macaques) lack the specific PERV receptor, HuPAR1, that is fully functional in human cells, instead containing a variant receptor, PAR1 (109Ser-Leu), which allows limited infection. Others have shown that PERV can infect rhesus macaques but do not seem to replicate following infection [41]. Another potential issue is that the short survival time after transplantation may affect PERV propagation after infection. Interestingly, PERV-A/C adapted to human cells has been shown to infect NHP cells due to mutations in long-terminal repeats that play important roles in viral replication [10,42]. Previous studies have reported that humans or NHPs exposed to porcine tissues after xenotransplantation have no productive infection in vivo [43,44,45]. The detection of PERV attributed to porcine cell microchimerism have reported no evidence of viral replication in host cells [40]. Therefore, our data suggest that PERV detected in NHP bladders were no evidence of productive infection. However, further studies are needed with long-term follow-up observations to ensure continued identification of infection by variant PERV.

In conclusion, we detected no transmission of PERV in heart xenotransplant tissues while PERV-A, B, and C were detected in the NHP bladder following kidney xenotransplantation. Encouragingly, PERV did not integrate into the host chromosome following renal transplantation, supporting further investigations in clinical trials. However, these results do not guarantee that PERV is not transmitted to the host. Our pre-clinical studies on NHPs are expected to provide valuable data on PERV transmission during xenotransplantation. All available methods should be used to effectively monitor PERVs and extreme caution exercised at all stages of control. To attain a higher level of safety in xenotransplantation, both extensive monitoring and inactivation of PERV are essential steps.

## Figures and Tables

**Table 1:** Information about pigs for xenotransplantation.

Type	Transgenic Pig	Rhesus Macaques	Survival Date (After Transplantation, Day)
Heart	GT-MCP/-MCP ^1^	NHP23-16	60
Heart	GT-CD39/-CD39 ^2^	NHP20-01	18
Kidney	GT-CD39/-CD39	NHP20-06	32
Kidney	GT-CD39/-CD39	NHP23-30	25

^1^ GT knockout transgenic pigs expressing MCP; ^2^ GT knockout transgenic pigs expressing CD39.

**Table 2:** Primers used for PCR and RT-PCR.

Name	Sequence (5′ to 3′)
PERV-*pol*-F	GATGAGCGTAAGGGAGTAGC
PERV-*pol*-R	TGCTTCCGTCAGTGAACCAG
PERV-*gag*-F	CCCGATCAGGAGCCCTATATCCTTACGTG
PERV-*gag*-R	CGCAGCGGTAATGTCGCGATCTCGT
PERV LTR 1-F	ATGCCCCCGAATTCCAGA
PERV LTR 1-R	GGTTAGGTTGCATTTTCATCCTT
PERV LTR 2-F	CCCCGAATTCCAGACCCT
PERV LTR 2-R	AGGTTGCATTTTCATCCTTTCATT
Porcine cytochrome B-F	CATTGGAGTAGTCCTACTATTTCCG
Porcine cytochrome B-R	CATTGGAGTAGTCCTACTATTTCCG
18sRNA-F	GTTCCGACCATAAACGATGCC
18sRNA-R	TGGTGGTGCCCTTCCGTCAAT

**Table 3:** Identification of PERV insertion of NHP’s bladder using inverse PCR.

Junction Sequence ^1^	Position	Species
ATGCCCCCGAATTCCAGA *CCCTGTTCCCTATAGGTAAAAGATCATGGTACTTAGACAGCA*	LOC110259374	Sus scrofa
ATGCCCCCGAATTCCAGA *CCCTGCTCCCTGCCAATAAATAGGTAGAAGGTCACACTTCTT*	CH242-417C1 on chromosome 4	Pig
ATGCCCCCGAATTCCAGA *CCCTGCTCCCTGCCAGTAAATCGGTAGAAGGTCACACTTCT*	LOC110261659	Sus scrofa
ATGCCCCGAATTCCAGA *CCCTGTTCCCTATAGGTAAAAGATCATGGTACTTAGACAGCAG*	LOC110256025	Sus scrofa
ATGCCCCGAATTCCAGA *TCCTTTCATTCCCCACTTCTTCTCTTGTTAATAGTTCTAA*	LOC110261658	Sus scrofa
ATGCCCCGAATTCCAGA *TACCAAGGCCTTCCGAGCTAAGGAGAAACTGACCTTTAGCCT*	CH242-160D12 on chromosome X	Sus scrofa

^1^ The underline is the LTR nucleotide, and the italic is cellular nucleotide.

## Supplementary Materials

The following are available online at https://www.mdpi.com/1999-4915/11/9/801/s1. Figure S1. Detection of PERV by PCR with type-specific primers from gDNA; Table S1. Primers used for detection of PERV types.

## References

[1] Griesemer A.D., Hirakata A., Shimizu A., Moran S., Tena A., Iwaki H., Ishikawa Y., Schule P., Arn J.S., Robson S.C. (2009). Results of Gal-Knockout Porcine Thymokidney Xenografts. Am. J. Transplant..

[2] Yamada K., Yazawa K., Shimizu A., Iwanaga T., Hisashi Y., Nuhn M., O’Malley P., Nobori S., Vagefi P.A., Patience C. (2005). Marked prolongation of porcine renal xenograft survival in baboons through the use of alpha 1,3-galactosyltransferase gene-knockout donors and the cotransplantation of vascularized thymic tissue. Nat. Med..

[3] Michel S.G., Madariaga M.L.L., Villani V., Shanmugarajah K. (2015). Current progress in xenotransplantation and organ bioengineering. Int. J. Surg..

[4] Mou L.S., Chen F.J., Dai Y.F., Cai Z.M., Cooper D.K.C. (2015). Potential alternative approaches to xenotransplantation. Int. J. Surg..

[5] Cooper D.K.C., Gaston R., Eckhoff D., Ladowski J., Yamamoto T., Wang L., Iwase H., Hara H., Tector M., Tector A.J. (2018). Xenotransplantation-the current status and prospects. Br. Med. Bull..

[6] Hryhorowicz M., Zeyland J., Slomski R., Lipinski D. (2017). Genetically Modified Pigs as Organ Donors for Xenotransplantation. Mol. Biotechnol..

[7] Denner J. (2016). How Active Are Porcine Endogenous Retroviruses (PERVs)?. Viruses-Basel.

[8] Lopata K., Wojdas E., Nowak R., Lopata P., Mazurek U. (2018). Porcine Endogenous Retrovirus (PERV) - Molecular Structure and Replication Strategy in the Context of Retroviral Infection Risk of Human Cells. Front. Microbiol..

[9] Denner J. (2017). Can Antiretroviral Drugs Be Used to Treat Porcine Endogenous Retrovirus (PERV) Infection after Xenotransplantation?. Viruses-Basel.

[10] Karlas A., Irgang M., Votteler J., Specke V., Ozel M., Kurth R., Denner J. (2010). Characterisation of a human cell-adapted porcine endogenous retrovirus PERV-A/C. Ann. Transplant.

[11] Deng Y.M., Tuch B.E., Rawlinson W.D. (2000). Transmission of porcine endogenous retroviruses in severe combined immunodeficient mice xenotransplanted with fetal porcine pancreatic cells. Transplantation.

[12] van der Laan L.J.W., Lockey C., Griffeth B.C., Frasier F.S., Wilson C.A., Onions D.E., Hering B.J., Long Z.F., Otto E., Torbett B.E. (2000). Infection by porcine endogenous retrovirus after islet xenotransplantation in SCID mice. Nature.

[13] Martina Y., Marcucci K.T., Cherqui S., Szabo A., Drysdale T., Srinivisan U., Wilson C.A., Patience C., Salomon D.R. (2006). Mice transgenic for a human porcine endogenous retrovirus receptor are susceptible to productive viral infection. J. Virol..

[14] Bittmann I., Mihica D., Plesker R., Denner J. (2012). Expression of porcine endogenous retroviruses (PERV) in different organs of a pig. Virology.

[15] Ericsson T.A., Takeuchi Y., Templin C., Quinn G., Farhadian S.F., Wood J.C., Oldmixon B.A., Suling K.M., Ishii J.K., Kitagawa Y. (2003). Identification of receptors for pig endogenous retrovirus. Proc. Natl. Acad. Sci. USA.

[16] Pierson R.N., Dorling A., Ayares D., Rees M.A., Seebach J.D., Fishman J.A., Hering B.J., Cooper D.K.C. (2009). Current status of xenotransplantation and prospects for clinical application. Xenotransplantation.

[17] Higginbotham L., Mathews D., Breeden C.A., Song M.Q., Farris A.B., Larsen C.P., Ford M.L., Lutz A.J., Tector M., Newell K.A. (2015). Pre-transplant antibody screening and anti-CD154 costimulation blockade promote long-term xenograft survival in a pig-to-primate kidney transplant model. Xenotransplantation.

[18] Iwase H., Hara H., Ezzelarab M., Li T., Zhang Z.Q., Gao B.S., Liu H., Long C., Wang Y., Cassano A. (2017). Immunological and physiological observations in baboons with life-supporting genetically engineered pig kidney grafts. Xenotransplantation.

[19] Shin J.S., Kim J.M., Kim J.S., Min B.H., Kim Y.H., Kim H.J., Jang J.Y., Yoon I.H., Kang H.J., Kim J. (2015). Long-term control of diabetes in immunosuppressed nonhuman primates (NHP) by the transplantation of adult porcine islets. Am. J. Transplant.

[20] Mohiuddin M.M., Singh A.K., Corcoran P.C., Thomas M.L., Clark T., Lewis B.G., Hoyt R.F., Eckhaus M., Pierson R.N., Belli A.J. (2016). Chimeric 2C10R4 anti-CD40 antibody therapy is critical for long-term survival of GTKO.hCD46.hTBM pig-to-primate cardiac xenograft. Nat. Commun..

[21] Kim N., Choi J., Kim S., Gwon Y.D., Cho Y., Yang J.M., Oh Y.K., Kim Y.B. (2016). Transmission of Porcine Endogenous Retrovirus Produced from Different Recipient Cells In Vivo. Plos ONE.

[22] Sandrin M.S., McKenzie I.F.C. (1994). Gal-alpha(1,3)gal, the major xenoantigen(s) recognized in pigs by human natural antibodies. Immunol. Rev..

[23] Cooper D.K.C., Koren E., Oriol R. (1994). Oligosaccharides and discordant xenotransplantation. Immunol. Rev..

[24] Hisashi Y., Yamada K., Kuwaki K., Tseng Y.L., Dor F., Houser S.L., Robson S.C., Schuurman H.J., Cooper D.K.C., Sachs D.H. (2008). Rejection of Cardiac Xenografts Transplanted from alpha 1,3-Galactosyltransferase Gene-Knockout (GalT-KO) Pigs to Baboons. Am. J. Transplant..

[25] Dwyer K.M., Robson S.C., Nandurkar H.H., Campbell D.J., Gock H., Murray-Segal L.J., Fisicaro N., Mysore T.B., Kaczmarek E., Cowan P.J. (2004). Thromboregulatory manifestations in human CD39 transgenic mice and the implications for thrombotic disease and transplantation. J. Clin. Invest..

[26] Imai M., Takigami K., Guckelberger O., Kaczmarek E., Csizmadia E., Bach F.H., Robson S.C. (2000). Recombinant adenoviral mediated CD39 gene transfer prolongs cardiac xenograft survival. Transplantation.

[27] Cai M., Huttinger Z.M., He H., Zhang W.Z., Li F., Goodman L.A., Wheeler D.G., Druhan L.J., Zweier J.L., Dwyer K.M. (2011). Transgenic over expression of ectonucleotide triphosphate diphosphohydrolase-1 protects against murine myocardial ischemic injury. J. Mol. Cell. Cardiol..

[28] Loveland B.E., Milland J., Kyriakou P., Thorley B.R., Christiansen D., Lanteri M.B., van Regensburg M., Duffield M., French A.J., Williams L. (2004). Characterization of a CD46 transgenic pig and protection of transgenic kidneys against hyperacute rejection in non-immunosuppressed baboons. Xenotransplantation.

[29] Fischer K., Kraner-Scheiber S., Petersen B., Rieblinger B., Buermann A., Flisikowska T., Flisikowski K., Christan S., Edlinger M., Baars W. (2016). Efficient production of multi-modified pigs for xenotransplantation by ‘combineering’, gene stacking and gene editing. Sci. Rep..

[30] Jensen T.S., Bjorge L., Wollen A.L., Ulstein M. (1995). Identification of the complement regulatory proteins cd46, cd55, and cd59 in human fallopian-tube, endometrium, and cervical mucosa and secretion. Am. J. Reprod. Immunol..

[31] Ko N., Lee J.W., Hwang S.S., Kim B., Ock S.A., Lee S.S., Im G.S., Kang M.J., Park J.K., Oh S.J. (2013). Nucleofection-Mediated 1,3-galactosyltransferase Gene Inactivation and Membrane Cofactor Protein Expression for Pig-to-Primate Xenotransplantation. Anim. Biotechnol..

[32] Hwang S., Oh K.B., Kwon D.J., Ock S.A., Lee J.W., Im G.S., Lee S.S., Lee K., Park J.K. (2013). Improvement of Cloning Efficiency in Minipigs Using Post-thawed Donor Cells Treated with Roscovitine. Mol. Biotechnol..

[33] Choi K., Shim J., Ko N., Eom H., Kim J., Lee J.W., Jin D.I., Kim H. (2017). Production of heterozygous alpha 1,3-galactosyltransferase (GGTA1) knock-out transgenic miniature pigs expressing human CD39. Transgenic Res..

[34] Kim H., Chee H.K., Yang J., Hwang S., Han K.H., Kang J., Park J.H., Kim J.S., Lee S.J., Ock S.A. (2014). Outcomes of Alpha 1,3-GT-knockout Porcine Heart Transplants Into a Preclinical Nonhuman Primate Model (vol 45, pg 3085, 2013). Transplant Proc..

[35] Lee D., Lee J., Kim H., Park H., Kim Y. (2007). Detection and Classification of Porcine Endogenous Retroviruses by Polymerase Chain Reaction. J. Anim. Sci. Technol..

[36] Soncini M., Signoroni P.B., Bailo M., Zatti D., Gregori A., Lombardi G., Albertini A., Wengler G.S., Parolini O. (2006). Use of highly sensitive mitochondrial probes to detect microchimerism in xenotransplantation models. Xenotransplantation.

[37] Morozov V.A., Wynyard S., Matsumoto S., Abalovich A., Denner J., Elliott R. (2017). No PERV transmission during a clinical trial of pig islet cell transplantation. Virus. Res..

[38] Yamada K., Sykes M., Sachs D.H. (2017). Tolerance in xenotransplantation. Curr. Opin. Organ Transpl..

[39] Moscoso I., Hermida-Prieto M., Manez R., Lopez-Pelaez E., Centeno A., Diaz T.M., Domenech N. (2005). Lack of cross-species transmission of porcine endogenous retrovirus in pig-to-baboon xenotransplantation with sustained depletion of anti-alpha Gal antibodies. Transplantation.

[40] Issa N.C., Wilkinson R.A., Griesemer A., Cooper D.K.C., Yamada K., Sachs D.H., Fishman J.A. (2008). Absence of Replication of Porcine Endogenous Retrovirus and Porcine Lymphotropic Herpesvirus Type 1 with Prolonged Pig Cell Microchimerism after Pig-to-Baboon Xenotransplantation. J. Virol..

[41] Denner J. (2018). Why was PERV not transmitted during preclinical and clinical xenotransplantation trials and after inoculation of animals?. Retrovirology.

[42] Gola J., Mazurek U. (2014). Detection of porcine endogenous retrovirus in xenotransplantation. Reprod. Biol..

[43] Specke V., Langford G., Schuurman H.J., Coulibaly C., Plesker R., Kurth R., Denner J. (2001). Porcine endogenous retroviruses (PERVs): Studies on transmission in vitro and inoculation of small animals and non-human primates in vivo. Xenotransplantation.

[44] Dinsmore J.H., Manhart C., Raineri R., Jacoby D.B., Moore A. (2000). No evidence for infection of human cells with porcine endogenous retrovirus (PERV) after exposure to porcine fetal neuronal cells. Transplantation.

[45] Denner J., Specke V., Tacke S., Schwendemann J., Coulibaly C., Plesker R., Kurth R., Langford G., Schuuman H.J. (2001). PERVs: Diagnostics, adaptation to human cells, but no transmission to small animals, non-human primates and man. Xenotransplantation.

