# Supplementary material for: Detection of Pig Cells Harboring Porcine Endogenous Retroviruses in Non-Human Primate Bladder After Renal Xenotransplantation
Source: Viruses. 2019 Aug 29;11(9):801. doi: 10.3390/v11090801 (PMC6784250; doi:10.3390/v11090801)
Supplement: Supplementary file 1 [file viruses-11-00801-s001.pdf]

Supplementary Materials

S1. Detection and classification of PERV by PCR

gDNA was isolated from different tissues with a DNA mini kit (Qiagen, CA, USA) and 50 ng of gDNA was used in PCR. The cycling reaction was performed under the following conditions: denaturation at 94℃ for 3 min, 30 cycles at 94℃ for 10 sec, 55℃ for 10 sec, 72℃ for 10 sec, and extension at 72℃ for 30 sec.

Table S1. Primers used for detection of PERV types

Name	Sequence (5′ to 3')
PERV-A-F	TCTGGGAGAAAGAAAGGATCTG
PERV-A-R	CGATTAAAGGCTTCAGTCTGGT
PERV-B-F	GGGCAAGTACAAAGTGATGAAA
PERV-B-R	TTCTAGGCGTGTTGGTAGGAAT
PERV-C-F	TGGATTAGAACTGGAAGC
PERV-C-R	TTTGACCCGTCAAGACCG

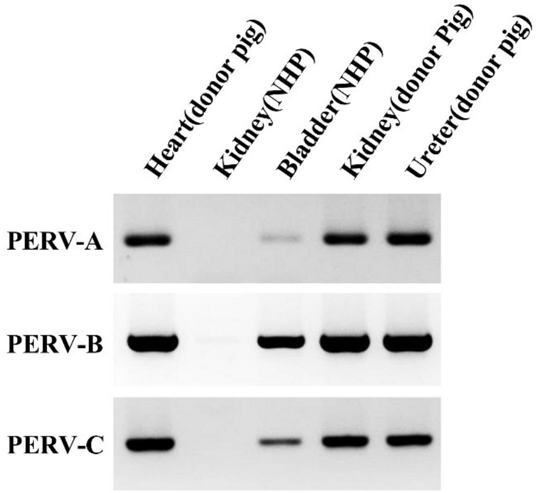

Figure S1. Detection of PERV by PCR with type-specific primers from gDNA. The PERV-subtypes (PERV-A, B, C) were detected by PCR with type-specific primers from gDNA of different tissues.
